# Latin American women’s experiences with medical abortion in settings where abortion is legally restricted

**DOI:** 10.1186/1742-4755-9-34

**Published:** 2012-12-22

**Authors:** Nina Zamberlin, Mariana Romero, Silvina Ramos

**Affiliations:** 1 Center for the Study of State and Society (CEDES) (External Researcher) and Adolescent Health Foundation (FUSA), Buenos Aires, Argentina; 2 Center for the Study of State and Society and National Scientific and Technical Research Council (CONICET), Buenos Aires, Argentina

**Keywords:** Medical abortion, Misoprostol, Latin America

## Abstract

Abortion is legally restricted in most of Latin America where 95% of the 4.4 million abortions performed annually are unsafe.

Medical abortion (MA) refers to the use of a drug or a combination of drugs to terminate pregnancy. Mifepristone followed by misoprostol is the most effective and recommended regime. In settings where mifepristone is not available, misoprostol alone is used.

Medical abortion has radically changed abortion practices worldwide, and particularly in legally restricted contexts. In Latin America women have been using misoprostol for self-induced home abortions for over two decades.

This article summarizes the findings of a literature review on women’s experiences with medical abortion in Latin American countries where voluntary abortion is illegal.

Women’s personal experiences with medical abortion are diverse and vary according to context, age, reproductive history, social and educational level, knowledge about medical abortion, and the physical, emotional, and social circumstances linked to the pregnancy. But most importantly, experiences are determined by whether or not women have the chance to access: 1) a medically supervised abortion in a clandestine clinic or 2) complete and accurate information on medical abortion. Other key factors are access to economic resources and emotional support.

Women value the safety and effectiveness of MA as well as the privacy that it allows and the possibility of having their partner, a friend or a person of their choice nearby during the process. Women perceive MA as less painful, easier, safer, more practical, less expensive, more natural and less traumatic than other abortion methods. The fact that it is self-induced and that it avoids surgery are also pointed out as advantages. Main disadvantages identified by women are that MA is painful and takes time to complete. Other negatively evaluated aspects have to do with side effects, prolonged bleeding, the possibility that it might not be effective, and the fact that some women eventually need to seek medical care at a hospital where they might be sanctioned for having an abortion and even reported to the police.

## Abstract (es)

**Abstract (Spanish):**

El aborto está legalmente restringido en la mayoría de América Latina donde 95% de los 4.4 millones de abortos que se realizan anualmente son inseguros.

El aborto con medicamentos es el uso de una droga o una combinación de drogas para interrumpi el embarazo. Mifepristona seguida de misoprostol constituye el regimen más efectivo y recomendado. En los lugares donde no está disponible la mifepristona, se utiiza misoprostol solo.

El aborto con medicamentos ha transformado radicalmente la práctica del aborto a nivel mundial, y particularmente en los contextos legalmente restrictivos. En América Latina, desde hace más de dos décadas, las mujeres utilizan el misoprostol para autoinducirse abortos.

Este artículo resume los hallazgos de una revisión bibliográfica sobre las experiencias de las mujeres con el aborto con medicamentos en países latinoamericanos donde el aborto voluntario es ilegal.

Las experiencias personales de las mujeres con el aborto con medicamentos son diversas y varían según el contexto, la edad, la historia reproductiva, el nivel socioeconómico y el conocimiento acerca del aborto con medicamentos así como las circunstancias físicas, emocionales y sociales que rodean a embarazo. Pero fundamentalmente, las experiencias están determinadas por la posibilidad de las mujeres de acceder a: 1) un aborto clandestino realizado bajo supervisión médica, o 2) información completa y precisa acerca del aborto con medicamentos. Otros factores clave incluyen el acceso a recursos económicos y el apoyo emocional.

Las mujeres valoran la seguridad y efectividad del aborto con medicamentos así como la privacidad que ofrece y la posibilidad de tener cerca a su pareja, un/a amiga/o, o persona de su confianza durante el proceso. Las mujeres perciben al aborto con medicamentos como menos doloroso, más fácil, más seguro, más práctico, menos costoso, más natural y menos traumático que otros métodos abortivos. Que sea auto-inducido y que evite el procedimiento quirúrgico también son señalados como ventajas. Las principales desventajas identificadas son que es doloroso y que lleva tiempo para que se complete. Otros aspectos evaluados negativamente incluyen los efectos secundarios, el sangrado prolongado, la posibilidad de que no sea efectivo, y el hecho de que algunas mujeres eventualmente deban solicitar atención médica en una institución donde sean sancionadas por haberse practicado un aborto y hasta denunciadas a la policía.

## Background

Only 28% of countries –most of them in the developed world– permit abortion upon request.^a ^Unsafe abortion and related mortality are both highest in countries with narrow grounds for legal abortion. An estimated 21.6 million unsafe abortions took place worldwide in 2008, almost all in developing countries, resulting in 47,000 maternal deaths and millions of women suffering injury, illness and lifelong disability [1].

In Latin America, 95% of the 4.4 million abortions performed annually are unsafe. Only Cuba, Mexico City, Uruguay and several Caribbean islands have liberal abortion laws [2]. The World Health Organization (WHO) estimates that in 2008, 12% of all maternal deaths in the region (1,100 in total) were due to unsafe abortion and about one million women were hospitalized for treatment of complications from unsafe abortion [1,2].

Despite legal restrictions, abortion is widely performed. When faced with an unwanted pregnancy many women will seek an abortion regardless of its legality. The safety of a clandestine procedure depends on the conditions under which it is performed which are primarily determined by the woman’s socioeconomic status. Women living in vulnerable social conditions who cannot afford safe clandestine abortions often turn to risky methods like the insertion of foreign bodies into the uterus, drinking toxic solutions, or procedures performed by unskilled providers [3,4]. Social and cultural beliefs against abortion as well as stigma are other barriers to safe abortion that make women turn to unsafe methods [5]. In addition, fear of ill treatment and legal reprisals might prevent women from seeking prompt medical care after an abortion [1,6].

Medical abortion (MA) refers to the use of a drug or a combination of drugs to terminate pregnancy. Mifepristone followed by misoprostol is the most effective and recommended regime. In settings where mifepristone is not available, misoprostol alone is used.^b ^Mifepristone is an anti-progestin that blocks the action of progesterone, a hormone necessary to maintain a pregnancy, and alters the endometrium causing the uterine lining to shed. Misoprostol is an analog of prostaglandin E_1 _that causes the cervix to soften and the uterus to contract, resulting in the expulsion of the uterine contents [7]. Until 63 days of gestation WHO recommends 200 mg of miefepristone administered orally followed by 800 mcg of misoprostol administered vaginally, buccally or sublingually 24 to 48 hours following ingestion of mifepristone [8]. In the case of misoprostol alone, WHO recommends 800 mcg of misoprostol administered by vaginal or sublingual routes, up to three repeat doses at intervals of at least three hours [8].

Fist trimester medical abortion is a highly safe and effective procedure [8]. Up to 9 weeks gestation effectiveness is 98% for the combined regime and between 75% and 90% for misoprostol alone [7-9].

The effects of medical abortion are similar to those associated with spontaneous abortion and include uterine cramping and prolonged bleeding. Common side effects include nausea, vomiting and diarrhea [7-9]. In 2005, the World Health Organization added mifepristone and misoprostol to its List of Essential Medicines^c ^for countries where abortion is not against the law. In 2009 misoprostol was also included for the treatment of incomplete abortion.

Medical abortion has radically changed abortion practices worldwide, and particularly in legally restricted contexts. Women can now access a non-invasive, safe and effective method, which is more affordable than surgical methods and does not require third party participation in the procedure [9,10]. In Latin America women have been using misoprostol for self-induced home abortions for over two decades [11]. As misoprostol became more widely used the use of highly unsafe and invasive abortion methods gradually became less frequent [2,12,13].

While mifepristone is unlikely to be available in countries with restrictive abortion laws, misoprostol is available in most of Latin America. It is generally approved to prevent gastric ulcers and not for gynecological and obstetric indications, except for four countries in the region^d ^which have it registered for some obstetric indication [14,15]. It is usually available in 200 mcg oral tablets and in some countries it is associated with an anti-inflammatory. It has been available in pharmacies since the late 1980s^e ^[16].

Information about medical abortion spreads mostly by word of mouth and through the Internet [17-19]. Pharmacies usually dispense misoprostol despite the fact that regulations in most countries require that it be sold only under prescription [20-23]. Medical abortion drugs are also accessed through providers in informal settings or on the Internet [19,24,25]. Local and international women’s groups and NGOs also disseminate information on medical abortion through the Internet, printed materials and hotlines that provide instructions on how to self-perform a medical abortion [19]. “Women on web”, an international digital community, provides on-line medical abortion services in different languages to women living in countries where there are no safe abortion services. Women complete a medical consultation through an interactive web-based questionnaire and if there are no contraindications they are sent a MA kit (mifepristone + misoprostol) by postal mail.^f ^In Argentina, Chile, Peru, Ecuador, Venezuela and Mexico there are abortion hotlines that provide information on how to use misoprostol for early pregnancy termination based on scientific information published by the World Health Organization and the Latin American Federation of OBGyN Societies (FLASOG).

Some NGOs in legally restrictive settings have clinics that provide information, counseling, medication and health care services at low cost or no cost to women seeking abortions. Some of these organizations offer both medical and surgical abortions performed by physicians, while others are run by trained non-medical counselors who provide information as well as the medication for the woman to administer herself, and follow up services [17,18,20,26].

In several Latin American countries medical abortion has enabled the implementation of harm reduction policies. Based on the right to health, autonomy, confidentiality and information, health professionals provide women with unwanted pregnancies pre-abortion counseling including information on how to self-induce a medical abortion, and postabortion care. Medication is not provided since it would be against the law, women have to obtain it by their own means. This strategy proved highly effective to prevent abortion related maternal deaths [27].

There is evidence that shows that in countries where voluntary abortion is not legal, increased use of medical abortion over other methods has increased the safety of self-induced procedures by reducing complications related to unsafe abortion [12,13,28-32]. Misoprostol and mifepristone are valuable resources that have great potential to expand access to safe abortion and therefore reduce maternal morbidity and mortality. However, the efficacy and safety of MA in legally restricted settings depends strongly on its adequate use in terms of dosage and gestational age, and the availability and accessibility of quality postabortion care services [12,13].

Globally, most research on medical abortion is situated in legal abortion contexts and focuses on trials of medication regimes and cost-effectiveness analyses of medical vs surgical abortion. Less attention has been paid to women’s experiences, perspectives and preferences [33]. Given the importance of medical abortion in reshaping the nature of abortion in legally restrictive settings, it is crucial to have a deep understanding of women’s experiences with this method.

This article summarizes the findings of a literature review on women’s experiences with medical abortion in Latin American countries where voluntary abortion is illegal. It includes studies focused on women’s perceptions regarding knowledge, access and use of medical abortion as well as the physical and subjective experience of undergoing a clandestine medical abortion. The article has a dual aim: to consolidate available evidence about the experiences of women who have a MA, and to foster the political and academic debate around unsafe abortion in Latin America and the key role that MA has in making abortion safer.

## Methodology

**Figure 1:**
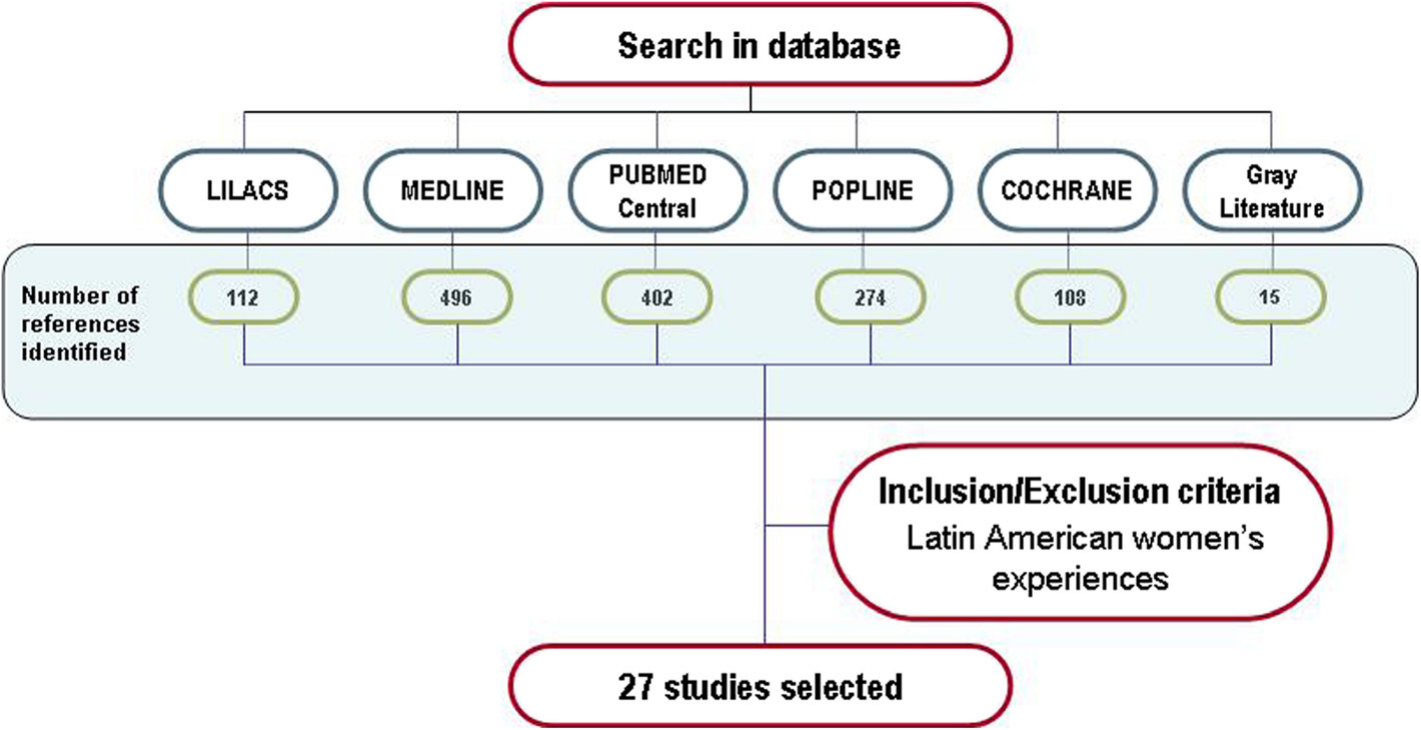
Summary of the findings of database search.

The review focused on studies produced in Latin America since 1990 to June 2011. Evidence in Spanish, Portuguese and English was identified through two different methodologies: 1) automatic searches of data bases (Lilacs,^g ^Medline,^h ^Pubmed Central,^i ^Popline^j ^and Cochrane^k^). 2) Gray literature search for unpublished or non-peer-reviewed sources by contacting key institutions that conduct research on abortion issues in the region, reviewing congresses abstracts, meeting summaries and news bulletins from pertinent networks.^l ^Keywords used include: abortifacient agents; misoprostol; methotrexate; mifepristone; abortion (abortion applicants; induced abortion; voluntary abortion; therapeutic abortion); interviews; stressful events; experiences; perception and opinion, connected by Boolean operators available in the different data bases. Additional file 1: Table S1 which can be accessed at http://cedes.org.ar/Rmw_oms_cedes_Table1.pdf includes details of keywords used, search strategies by data base and number of articles identified. Figure 1 shows the process of applying the selection criteria to the items identified in the search.

## Results

The literature review identified 27 articles/papers which are highly diverse in terms of methodological approaches, data collection techniques, design, inclusion criteria, and sample methods.^m ^Most studies were carried out between 2005 and 2011. Earlier production is scarce, except for the case of Brazil where over half a dozen articles were published in the early and mid 1990s. Brazil was the first country to report the use of misoprostol for self-induced clandestine abortions, and most research addresses women hospitalized for postabortion care [12,34-38].

Few studies focus exclusively on medical abortion, while the rest address medical abortion within a wide variety of issues related to the abortion event such as reasons for seeking an abortion, the decision making process, or attitudes towards abortion and include women who used medical abortion as well as women who resorted to other means of pregnancy termination. In these cases, we only selected the information which could shed light on the experience of undergoing a medical abortion.

Studies differ in the time elapsed between the abortion and the moment data was collected. Some only included women who had had an abortion in the previous six months or in the previous two years, while others encompass much longer periods of time, up to 20 years.

Selected studies can be classified in the following categories according to the study population and sample used as shown on Additional file 2: Table S2.

Studies include both qualitative and quantitative approaches that applied different data collection techniques: 1) in-depth interviews focused on the narrative of the abortion experience; 2) review of medical records and surveys of women hospitalized for postabortion care to describe socio-demographic characteristics, previous use of contraception, abortion method used and incidence of complications (these studies do not analyze in depth the experience around self-inducing the abortion); and 3) literature reviews.

Despite the diverse approaches, all articles address critical aspects of the medical abortion experience. The following section summarizes the main dimensions present in the evidence reviewed which include: knowledge and information about medical abortion; choice of method; obtaining the medication; the medical abortion process: the physical and the psychological experience.

### Knowledge and information about medical abortion

Women usually learn about MA when they have an unintended pregnancy. Previous knowledge is scarce and superficial, often limited to knowing about the existence of “abortion pills” that sometimes are confused with emergency contraception [17,30,39-41].

Deciding to have an abortion and doing so is not a linear process, particularly in legally restricted settings, not only because women might face ambivalence and personal, familial and social conflict, but also because they might take several different “small actions” which do not follow a sequential or organized pattern [42]. Faced with an unwanted pregnancy, women start searching for solutions that might eventually lead to a MA. Many find out about MA only after unsuccessfully trying other supposedly abortifacient methods such as herbal infusions and hormonal injectables [17,18,25,26,30,39,43].

Clandestinity implies that information about MA is not openly and publicly available but that it rather flows through hidden informal or “underground” channels. Female relatives, friends, neighbors and the sexual partner, are the ones who provide information or help to identify sources of information such as women who had abortions in the past or who have been close to women in a similar situation, women’s health organizations, health professionals, pharmacies and Internet sites [11,24,35,36,39,43,44].

Women who reach harm reduction services often find out about MA through the counseling provided [25]. In some cases men lead the search for information resorting to other men who can provide advice [12,45,46] while women adopt a more passive stance [47].

Information about MA is mostly spread by word of mouth and it is therefore highly diverse and fragmented, particularly when it comes from laypeople, but pharmacy staff and health professionals also provide highly heterogeneous information that in some cases differs significantly from scientific standards [19-21,23,30,39,44,48].

Complete and correct information about how to use and what to expect from MA, including dosage, routes of administration, mechanism of action, effectiveness, contraindications and side effects is crucial in determining the outcome of the process and the woman’s experience. Women who receive accurate and complete information on how the medical abortion process will develop and what is and what is not normal have more positive experiences. In this sense, counseling by a health care professional or a qualified counselor is vital for the whole abortion process [4,13,25,39].

### Choice of method

As with most abortion matters within legally restricted settings, the experience of undergoing a medical abortion is primarily determined by social class. Lower income women do not have an array of safe methods to choose from according to their needs and preferences [39,49], but they do perceive that using medication is safer than introducing objects or other traditional unsafe methods [12,24]. Preference for MA is also expressed based on fear of unsafe medical procedures or because they associate it more with a menstrual regulation process. However, cost and accessibility are key factors for deciding for a MA, more than personal preferences or a balance of its advantages and disadvantages. Women make pragmatic decisions regarding the abortion method based on their possibilities that are not necessarily “real choices” [17,18]. In the absence of financial constraints some would prefer a surgical abortion performed by a physician or a medically supervised procedure [35,39,49].

Women value the safety and effectiveness of MA as well as the privacy that it allows and the possibility of having their partner, a friend or a person of their choice nearby during the process. Women perceive MA as less painful, easier, safer, more practical, less expensive, more natural and less traumatic than other methods. The fact that it is self-induced and that it avoids surgery is also pointed out as an advantage [11,17,18,24,35,44].

Main disadvantages identified by women are that MA is painful and takes time to complete. Other negatively evaluated aspects have to do with side effects, prolonged bleeding, the possibility that it might not be effective, and the fact that some women eventually need to seek medical care at a hospital where they might be sanctioned for having an illegal abortion and even reported to the police [11,17,18].

### Obtaining the medication

In most cases women have to procure the medication by themselves, except those who access MA through an institution or a health professional that provides misoprostol. Risk reduction services offer information on medical abortion as a way to avoid higher risk methods but women must obtain the drug on their own. Some women feel highly frustrated when they learn that abortions are not practised nor is misoprostol provided by such services [25].

In many Latin American countries pharmacies are widely used as a source of medical advice, especially by lower income populations, and women have traditionally resorted to pharmacies in search for drugs to bring on menstruation when they have a delayed menstrual period [13,21]. Misoprostol is purchased at retail pharmacies either as the entire package or by the pill, usually without prescription despite the fact that government regulations require sale under prescription [11,20-22,34]. Pharmacy staff often recommend misoprostol for pregnancy termination but their knowledge about dosage, route of administration, side effects complications and effectiveness is often poor in quality [20,21]. They usually advise to seek medical care once bleeding starts [22].

Different levels of difficulty exist in obtaining misoprostol related to the local regulation of the drug and the level of government control over pharmacy sales. Stricter control makes access more difficult, pushing women to the black market where prices are higher [25,48,50].

In settings where misoprostol is sold only under prescription women display a variety of strategies to either obtain a prescription or to buy the drug without one. These include paying for a prescription, claiming that the drug is not for ObGyn purposes either by obtaining and presenting a prescription from a non-ObGyn specialist, or asking a man or an older woman to buy the drug for them arguing that is for their own use [22].

Internet is extensively used to search for information on MA and to a lesser extent to buy misoprostol. The quality and authenticity of misoprostol sold on the Internet by individuals with lucrative purposes is questionable since it is sometimes not provided in its original packaging and can be fake [19,50,51]. The studies reviewed do not report use of telemedicine websites or hotlines for obtaining MA medication among Latin American women.^n^

Prices vary widely, and depend on where the medication is purchased [16,39,40]. Even if medical abortion is considerably less expensive than surgical methods, it is still unaffordable for poor women and adolescents who do not have ready access to cash. Women implement different strategies to gather the money to buy the medication: borrowing money from friends and relatives, asking for a salary advance, working overtime, selling valuable objects [25,39,49]. One study even refers to women travelling or contacting people in neighboring countries to obtain the medication [39].

Sometimes women who bought the whole package and have pills left after completing the abortion either offer them or sell them to other women in need of misoprostol as a way of female solidarity or cost-recovery [22,39].

Obtaining the drug implies not just having the money but entering the circuit of irregular sale of misoprostol. Internet is the main source of this kind of information.

Accessing the drug will depend on the woman’s economic capacity, social network, personal skills, and support from others. Male partners who are involved in the abortion decision usually have an active role in obtaining the medication, particularly in contributing economically and searching for where to buy it [12,25,46].

### The medical abortion process: the physical experience

Like the majority of abortions, most medical abortions take place within the first 12 weeks of pregnancy [11,12,32,39,40]. Women generally understand that earlier abortions are safer, but sometimes the abortion is delayed because time is spent seeking information, trying ineffective methods, searching for the medication and raising the money to buy it [30,39]. Second and even third trimester self-induced home abortions with misoprostol have been reported [11,34]. These situations can be extremely risky.

For most women, getting ready for a medical abortion means preparing for something unknown, an unexperienced event that can trigger unexpected consequences [39,49]. They must decide when and where to do it, if someone will be with them at that moment and who they want that person to be. If they have children they must arrange for someone to look after them and organize the domestic chores [17,18,39].

Women appreciate the privacy that medical abortion allows them. In some circumstances they conceal the abortion from other people. There are testimonies of women who went through a MA without altering their daily lives and surrounded by relatives, or even their partner, who were unaware of their condition. In other cases, hiding the abortion from other household members is difficult and problematic [17,26,44].

Women often prefer to use the pills during the night as they perceive it to be safer, with few chances of being interrupted, and they are usually at home while others are resting [12,17,39]. The night might also feel like a more private and protected time for doing something illegal [39].

It is not uncommon for women to use misoprostol together with other methods, mostly ineffective ones like teas and other infusions, and injections bought in pharmacies [12,51,52].

Few of the women who obtain the medication outside clinical settings can specify the name of the medication they used for pregnancy termination [17,39,40,44,49,53] and cannot precise if they were antibiotics, analgesics or tranquilizers [17].

The information that women receive outside clinical settings about how to use misoprostol is highly diverse, consequently, women use misoprostol in a variety of ways [24,35,51] with doses ranging between 4 and 16 tablets, and much higher doses in extreme cases [11,35,54,55]. However the median dose is usually 800 mcg, the adequate dosage for early abortion [34,35,37]. Intervals between doses also vary widely [24].

Women use misoprostol vaginally, orally or a combination of both routes [24,35-37] and seem to prefer oral rather than vaginal administration [13]. Some women dislike or are uncomfortable with the vaginal administration of tablets, or are not sure they can insert them correctly by themselves. Several authors relate oral preference to the idea of menstrual regulation, while vaginal insertion is culturally more linked to abortion [12,17].

After inserting or taking the pills women wait for something to happen. When possible, they stay home and rest. Others continue with their daily routines. In some cases expulsion or heavy bleeding took them by surprise while they were at school or at work [17,49].

Bleeding usually starts few hours after the first dose and is most abundant at 6 to 12 hours after insertion [44] but can also take much longer [24,35]. Bleeding can last between 1 and 60 days [24]. Several studies report testimonies of heavy bleeding, or bleeding more abundant than what women had expected [30,40]. Often women are unable to determine whether their symptoms are normal or abnormal or whether a complete abortion has occurred [30].

Common side effects include chills, diarrhea, nausea, headache, dizziness and fever. These are usually well tolerated [17,40]. Most women experience pain of different intensity and duration [24,40,44]. Generally the most severe pain takes place the first day after inserting/taking the pills, particularly after 5 to 7 hours, and later diminishes [44]. Some women report unbearable sustained pain for several hours [17,25,26]. Those who have medical supervision are recommended to take pain relief medication [17].

Some women start and finish the MA process at home. Others do not wait enough to complete the abortion alone and seek medical assistance in health care institutions where a surgical uterine evacuation procedure is usually performed. Some women seek medical care shortly after bleeding starts, either because they are afraid that something bad will happen to them, or because they were told to do so by the person who instructed them on how to use the medication [12,20,22,30,35,36,44,45].

As information on MA becomes more widespread and women gain more experience they make better use of misoprostol [11,35]. Evidence collected from hospitalized postabortion women shows that prevalence of severe complications is lower among women who used misoprostol than among those who used other methods [29,34,35].

In some cases attempts to terminate pregnancy with misoprostol are not successful and pregnancy continues. Women who access medical services that perform abortions can resort to a surgical abortion [17,18,26]. But women who lack this alternative are left with no options. The possibility of having a surgical procedure depends on their economic capacity. These are critical situations marked by anxiety and distress, particularly when women are aware of the possible teratogenic effects of the medication [39].

### The psychological experience

Having a medical abortion means a direct and vivid physical experience which triggers strong emotions, fantasies and fears. Few of the articles reviewed refer to the psychological aspects of the MA experience, which are closely related to the physical experience, the information the woman has received, and the availability or lack of medical and emotional support [17,18,39,45,49].

Some women relate MA to a menstrual regulation process or something akin to getting their period, which reduces emotional distress and helps them to cope with the process [11,17,18,44]. On the other hand, many women go through a MA feeling that it is an unknown process of which they have no full control [39]. Common feelings are fear of the negative reactions in the body, and concern linked to pain and bleeding. Women are very anxious about heavy vaginal bleeding and fear they can bleed to death or suffer long lasting health complications including infertility [25,43,51]. Women who have legal medical abortions in a medically controlled setting are less concerned about bleeding [56].

Testimonies also reflect uncertainty and anxiety about how long the process will last, when they will be able to return to their daily life and whether or not certain activities are safe to do (working, swimming, bathing, physical activity, sex) [17].

Women who have medical supervision and/or receive detailed information from a qualified source and know what to expect in terms of bleeding, pain and side effects report more positive experiences with less anxiety and fear, feel more in control and tend to remain calm [29]. In addition, previous experience with pregnancy and delivery seems to contribute to a better management of the situation [17].

Many women, but particularly those who undergo the process with no counseling or supervision, have emotionally draining experiences marked by fear of negative consequences, anxiety and concern. These are mostly elicited by the clandestine context and the lack of medical back-up in moments when women feel extremely vulnerable and out of control of the situation [39,45]. Some women refrain from seeking medical care out of fear that they will be mistreated, penalized or given medication to retain the pregnancy [43].

Affective support and company are vital during the MA process, particularly for adolescents who are more vulnerable than older women in the same situation [25,26]. Women are usually accompanied by their partner, female relatives (mother, sister) or female friends who help to minimize discomfort or simply stay by them [17,25,39,49]. Having the support of their close ones gives women not only the possibility of sharing doubts and fears, and not feeling alone -which is reassuring and helps them to remain calm- but also implies the possibility of accessing economic resources or having someone who will take care of their children if they have any [25,39]. Women who go through the process alone or conceal the abortion from other household members usually have emotionally difficult situations [17,25,47].

## Conclusions

Women’s personal experiences with medical abortion are diverse and vary according to context, age, reproductive history, social and educational level, knowledge about medical abortion, and the physical, emotional, and social circumstances linked to the pregnancy. But most importantly, experiences are determined by whether or not women have the chance to access: 1) a medically supervised abortion in a clandestine clinic, or 2) complete and accurate information on medical abortion**.** Other key factors are access to economic resources and emotional support.

The experiences of women who access medical abortion under clinical supervision or who receive qualified counseling vary significantly from those who use misoprostol on their own or with the help of laypeople. Women who access MA services provided by trained personnel have the most positive experiences. They are assured genuine medication, have medical backup in case of complications and can resort to a surgical procedure in case of medical abortion failure or incomplete abortion. Women who do not access this type of services are on their own during the whole process, including finding alternative solutions if the attempts with misoprostol are not successful. Their experiences are characterized by anxiety and fear of negative consequences to their health. Doing something illegal and acting in a clandestine way is a major cause of distress that makes women feel vulnerable and unprotected. The presence of significant others and social networks are helpful during the whole process.

Overall women find MA acceptable even though it might not be their first choice if they had the possibility to select between surgical or medical methods. The positive and negative attributes of MA perceived by women in legally restricted contexts are very similar to those expressed by women in legal abortion settings [33,57-59] The major difference lies in the confidence that legality provides and the reassurance that the heath system accompanies them and cares for them during the abortion process.

Women who have MAs in legally restricted settings access mostly misoprostol and they tend to use it in rather adequate doses. The literature reviewed over a 20 year period shows that recent studies report more proper use of misoprostol while older studies refer to more “anarchic use” including excessive doses. Also, most side effects described are similar to those reported in clinical trials [8]. This could be indicating that information on MA is becoming more widespread in the region.

The information on the psychological experience of women who have medical abortions in legally restricted settings does not allow to make comparisons with the abortion experience in general. However, using a self-induced method in the privacy of their homes seems to reduce the stress related to the illegal character of abortion.

Latin America has a long tradition of academic production and social research in sexual and reproductive health and a strong women’s movement mobilized around the abortion issue [60,61]. However, the literature review shows that there are few studies in the region that specifically focus on the medical abortion experience from the woman’s perspective. Furthermore, several countries have no research production on this field at all. Research on abortion in legally restricted settings implies additional ethical and logistical obstacles to a naturally socially sensitive issue which affect the possibility of exploring the issue and obtaining valid information, particularly when it involves approaching women who have had illegal abortions [61,62].

The evidence reviewed is highly heterogeneous and was produced by different approaches and methodologies; therefore a comparative analysis is not possible. Most available research is based on samples of women hospitalized after an abortion or women who accessed risk reduction services. Only a minority of studies include women who completed the MA process alone, without preabortion counseling and/or postabortion care. Adolescent women are underrepresented in the available studies and the experience of those under age 15 is completely absent. We know nothing about women who had failed medical abortions and continued on with their pregnancy, and very little about those who had a legal abortion within the health care system or those who received misoprostol for postabortion care. In addition, most literature comes from large urban settings, and few studies include rural or indigenous women [18]. More and updated scientific evidence on medical abortion in Latin American is needed in order for researchers, activists, policy makers and health care providers to have a better and more comprehensive understanding of its impact on women’s lives and health.

Given the results of this literature review, it is clear that the research agenda on women’s experiences with medical abortion is not yet fully developed in the Latin American region. More research is needed in order to get a comprehensive picture. Different subpopulations and issues need to be addressed and other study designs and methodologies implemented. The experience with medical abortion and its results and impact on adolescents, rural and indigenous women, and women accessing legal abortions within restrictive legal contexts need to be better understood. Issues such as the experience of successful use of medical abortion by women who use it with no medical supervision needs to be better assessed given the potential of medical abortion as a self-used technology. As for study designs, general population is still not in the picture and it should be. In order to overcome the limitations of the widely used convenience sampling methods, respondent-driven sampling (RDS) needs to be assessed as a probabilistic sampling strategy to study this specific hard-to-reach population [63]. Additionally, comparative analysis among countries needs also to be developed not only to understand the use of this new technology in different cultural and social contexts but also to get a more robust understanding of its impact on women’s experiences in legally and culturally restrictive environments.

Women in developing countries where abortion is legally restricted have a great need for safe, affordable and simple abortion methods. Misoprostol is a very important contribution which has facilitated women better access than ever before to an effective and safe method for early self-induced pregnancy termination. This has had a positive impact on their health. Women need to have access to detailed and complete information on MA through a wide array of communication channels, as well as pre and post abortion services to optimize the great potential that MA has to make abortions safer in legally restrictive settings.

## Endnotes

^a^98% of countries in the world allow abortion to save a woman’s life, most of them also permit one or several further conditions. However, in many settings legal abortions are hard to access and are rarely officially practiced [1].

^b^Methotrexate has also been used in combination with misoprostol as a medical method for early abortion in some countries where mifepristone is not available. However, a WHO toxicology panel recommended against the use of methotrexate for inducing abortion, based on concerns of teratogenicity if the method fails and the pregnancy is not interrupted [8].

^c^The WHO List of Essential Medicines contains those medicines which are considered to satisfy the priority health needs of the population of developing countries, and which have been selected on the basis of their efficacy, safety and cost-effectiveness.

^d^Brazil, Peru, Mexico [14] and Argentina [15].

^e^In the case of Brazil, in the late eighties misoprostol was available over the counter in pharmacies and became a popular abortifacient method. In 1991 the government severely restricted its sale and in some states it was completely banned. Currently it is sold exclusively for hospital use. However, misoprostol continues to be sold in the black market [16,34].

^f^In 2008 Gomperts *et al*. published a study based on 484 women from 33 different countries who contacted Women on Web and received a medical abortion kit (mifepristone + misprostol) by postal mail. Results show that women seem capable of self-administering MA when proper information and instructions are provided through Internet and additional interactive online consultations and email correspondence [64].

^g^Latin American and Caribbean Health Sciences Information.

^h^U.S. National Library of Medicine.

^i^U.S. National Institutes of Health.

^j^John's Hopkins Bloomberg School of Public Health.

^k^The Cochrane Collaboration.

^l^In 2009, the Latin American Consortium against Unsafe Abortion (CLACAI) carried out a literature review on Latin American women’s experiences with medical abortion coordinated by Nina Zamberlin which included designing a methodology for data collection [65]. In 2010 CLACAI created RepoCLACAI, a repository on abortion that systematizes research studies, technical documents and guidelines produced in the Latin American region (http://www.clacaidigital.info).

^m^Studies were not quality assessed and therefore none was excluded on such basis.

^n^Gomperts *et al*. analyze access and use to MA through Women on Web (http://www.womenonweb.org) by women in 33 different countries where abortion is legally restricted, but data is not disaggregated by country or region [64].

## Abbreviations

MA: Medical abortion; WHO: World Health Organization.

## Competing interests

The authors declare that they have no competing interests.

## Authors' contributions

NZ carried out the selection of studies based on the inclusion criteria, read, summarized and analyzed all the material, and wrote the draft version of the article. SR and MR established the criteria for the bibliographical search, discussed the findings and drafts, and contributed to the final article. All authors read and approved the final manuscript.

## Supplementary Material

Additional file 1 **Table S1.** Description of search strategies for identification of articles. Click here for file

Additional file 2 **Table S2.** Selected articles. Click here for file
