# Supplementary material for: Latin American women’s experiences with medical abortion in settings where abortion is legally restricted
Source: Reprod Health. 2012 Dec 22;9:34. doi: 10.1186/1742-4755-9-34 (PMC3557184; doi:10.1186/1742-4755-9-34)
Supplement: Additional file 1 — Table S1. Description of search strategies for identification of articles. [file 1742-4755-9-34-S1.docx]

**Table 1. Description of search strategies for identification of articles**

| **Database and search strategies** | **Number of articles identified** |
| --- | --- |
| **LILACS** (Latin American and Caribbean Health Sciences Information) | |
| ((“Abortion Applicants” OR “Abortion, Induced” OR “Abortion, Legal” OR  “Abortion, Therapeutic”) [Subject descriptor] AND “women” [Subject descriptor]) | 9 |
| (“misoprostol$”[Subject descriptor] AND “women” [Subject descriptor]) | 0 |
| (“misoprostol$”[Subject descriptor] AND “Interviews as Topic” [Subject descriptor]) | 1 |
| ((“Abortion Applicants” OR “Abortion, Induced” OR “Abortion, Legal” OR  “Abortion, Therapeutic”) [Subject descriptor] AND “Interviews as Topic” [Subject descriptor]) | 16 |
| (“misoprostol$”[Subject descriptor] AND (“Abortion Applicants” OR “Abortion, Induced” OR “Abortion, Legal” OR  “Abortion, Therapeutic”) [Subject descriptor]) | 18 |
| (“misoprostol$”[Subject descriptor] AND “Life Change Events” [Subject descriptor]) | 0 |
| ((“Abortion Applicants” OR “Abortion, Induced” OR “Abortion, Legal” OR  “Abortion, Therapeutic”) [Subject descriptor]AND “Life Change Events” [Subject descriptor]) | 0 |
| (“women” [Subject descriptor] AND “Life Change Events” [Subject descriptor]) | 9 |
| (“Abortifacient Agents” OR “Abortifacient Agents, Nonsteroidal” OR “Abortifacient Agents, Steroidal” [Subject descriptor]) | 48 |
| ((“Abortion Applicants” OR “Abortion, Induced” OR “Abortion, Legal” OR  “Abortion, Therapeutic”) [Subject descriptor] AND Mifepristone [Subject descriptor]) | 5 |
| ((“Abortion Applicants” OR “Abortion, Induced” OR “Abortion, Legal” OR  “Abortion, Therapeutic”) [Subject descriptor] AND Methotrexate [Subject descriptor]) | 6 |
| MEDLINE (U.S. National Library of Medicine) | |
| ((“Abortion Applicants” OR “Abortion, Induced” OR “Abortion, Legal” OR  “Abortion, Therapeutic”) [Subject descriptor] AND “women” [Subject descriptor]) | 96 |
| (“misoprostol$”[Subject descriptor] AND “women” [Subject descriptor]) | 2 |
| (“misoprostol$”[Subject descriptor] AND “Interviews as Topic” [Subject descriptor]) | 7 |
| ((“Abortion Applicants” OR “Abortion, Induced” OR “Abortion, Legal” OR  “Abortion, Therapeutic”) [Subject descriptor] AND “Interviews as Topic” [Subject descriptor]) | 109 |
| (((“misoprostol$”[Subject descriptor] AND (“Abortion Applicants” OR “Abortion, Induced” OR “Abortion, Legal” OR  “Abortion, Therapeutic”) [Subject descriptor]) AND (“women” [Subject descriptor] OR “Interviews as Topic” [Subject descriptor] OR “Life Change Events” [Subject descriptor])) | 7 |
| (“misoprostol$”[Subject descriptor] AND “Life Change Events” [Subject descriptor]) | 0 |
| ((“Abortion Applicants” OR “Abortion, Induced” OR “Abortion, Legal” OR  “Abortion, Therapeutic”) [Subject descriptor] AND “Life Change Events” [Subject descriptor]) | 16 |
| (((“Abortifacient Agents” OR “Abortifacient Agents, Nonsteroidal” OR “Abortifacient Agents, Steroidal” [Subject descriptor]) AND (“Abortion Applicants” OR “Abortion, Induced” OR “Abortion, Legal” OR  “Abortion, Therapeutic”) [Subject descriptor]) AND “women” [Subject descriptor]) | 6 |
| (((“Abortion Applicants” OR “Abortion, Induced” OR “Abortion, Legal” OR  “Abortion, Therapeutic”) [Subject descriptor] AND Mifepristone [Subject descriptor]) AND “women” [Subject descriptor]) | 19 |
| ((“Abortion Applicants” OR “Abortion, Induced” OR “Abortion, Legal” OR  “Abortion, Therapeutic”) [Subject descriptor] AND Methotrexate [Subject descriptor]) | 234 |

**Table 1. Description of search strategies for identification of articles (cont.)**

| **PUBMED Central** (U.S. National Institutes of Health) |  |
| --- | --- |
| (("misoprostol"[MeSH Major Topic] OR "misoprostol/adverse effects"[MeSH Major Topic] OR "misoprostol/economics"[MeSH Major Topic] OR "misoprostol/etiology"[MeSH Major Topic] OR "misoprostol/immunology"[MeSH Major Topic] OR "misoprostol/organization and administration"[MeSH Major Topic] OR "misoprostol/pharmacology"[MeSH Major Topic] OR "misoprostol/physiology"[MeSH Major Topic] OR "misoprostol/therapeutic use"[MeSH Major Topic]) AND ("women"[MeSH Major Topic] OR "women/physiology"[MeSH Major Topic] OR "women/psychology"[MeSH Major Topic] OR "women's health"[MeSH Major Topic])) | 9 |
| (("abortion applicants"[MeSH Major Topic] OR "abortion, induced"[MeSH Major Topic] OR "abortion, legal"[MeSH Major Topic] OR "abortion, habitual"[MeSH Major Topic] OR "abortion, therapeutic"[MeSH Major Topic] OR abortion[MeSH Major Topic]) AND ("Life Change Events "[MeSH Major Topic] OR "interviews as topic"[MeSH Major Topic)) | 28 |
| ((("abortion applicants"[MeSH Major Topic] OR "abortion, induced"[MeSH Major Topic] OR "abortion, legal"[MeSH Major Topic] OR "abortion, habitual"[MeSH Major Topic] OR "abortion, therapeutic"[MeSH Major Topic] OR abortion[MeSH Major Topic]) AND ("women"[MeSH Major Topic] OR "women/physiology"[MeSH Major Topic] OR "women/psychology"[MeSH Major Topic] OR "women's health"[MeSH Major Topic])) AND ("Life Change Events "[MeSH Major Topic] OR "interviews as topic"[MeSH Major Topic)) | 1 |
| ((("women"[MeSH Major Topic] OR "women/physiology"[MeSH Major Topic] OR "women/psychology"[MeSH Major Topic] OR "women's health"[MeSH Major Topic]) AND ("abortion applicants"[MeSH Major Topic] OR "abortion, induced"[MeSH Major Topic] OR "abortion, legal"[MeSH Major Topic] OR "abortion, habitual"[MeSH Major Topic] OR "abortion, therapeutic"[MeSH Major Topic] OR abortion[MeSH Major Topic])) AND Review$) | 23 |
| (("abortifacient agents"[MeSH Terms]) AND "abortifacient agents, nonsteroidal"[MeSH Terms]) AND "abortifacient agents, steroidal"[MeSH Terms]  AND ("women"[MeSH Major Topic] OR "women/physiology"[MeSH Major Topic] OR "women/psychology"[MeSH Major Topic] OR "women's health"[MeSH Major Topic])) | 2 |
| ((("abortifacient agents"[MeSH Terms]) AND "abortifacient agents, nonsteroidal"[MeSH Terms]) AND "abortifacient agents, steroidal"[MeSH Terms]  AND Review$) | 31 |
| (("mifepristone"[MeSH Terms] AND ("abortion applicants"[MeSH Major Topic] OR "abortion, induced"[MeSH Major Topic] OR "abortion, legal"[MeSH Major Topic] OR "abortion, habitual"[MeSH Major Topic] OR "abortion, therapeutic"[MeSH Major Topic] OR abortion[MeSH Major Topic])) AND Review$) | 103 |
| ("Methotrexate"[MeSH Terms]  AND ("abortion applicants"[MeSH Major Topic] OR "abortion, induced"[MeSH Major Topic] OR "abortion, legal"[MeSH Major Topic] OR "abortion, habitual"[MeSH Major Topic] OR "abortion, therapeutic"[MeSH Major Topic] OR abortion[MeSH Major Topic])) | 180 |
| (("Methotrexate"[MeSH Terms] AND ("abortion applicants"[MeSH Major Topic] OR "abortion, induced"[MeSH Major Topic] OR "abortion, legal"[MeSH Major Topic] OR "abortion, habitual"[MeSH Major Topic] OR "abortion, therapeutic"[MeSH Major Topic] OR abortion[MeSH Major Topic])) AND AND Review$) | 25 |

**Table 1. Description of search strategies for identification of articles (cont.)**

| **POPLINE** (John's Hopkins Bloomberg School of Public Health) | |
| --- | --- |
| (misoprostol & (perception / opinion)) | 2 |
| ((abortion & pharmacol*) | 4 |
| ((((abortion) & (perception / opinion)) & women) | 118 |
| ((misoprostol & abortion) & women) | 44 |
| ((abortion) & (abortifacient agents / steroid abortifacients)) | 2 |
| (RU-486 & abortion) | 29 |
| methotrexate | 53 |
| (mifepristone & abortion) | 22 |
| **Cochrane** (The Cochrane Collaboration) | |
| ((misoprostol and women) and experience) | 54 |
