# Supplementary material for: Latin American women’s experiences with medical abortion in settings where abortion is legally restricted
Source: Reprod Health. 2012 Dec 22;9:34. doi: 10.1186/1742-4755-9-34 (PMC3557184; doi:10.1186/1742-4755-9-34)
Supplement: Additional file 2 — Table S2. Selected articles. [file 1742-4755-9-34-S2.doc]

**Table 2.** **Selected articles**

| **N°** | **Citation** | **Country/ies** | **Studies based on:** | | | | | | | |
| --- | --- | --- | --- | --- | --- | --- | --- | --- | --- | --- |
|  |  |  | **Women who accessed private health services that provide medical abortion** | **Women who accessed (health) institutions that provide information about medical abortion (harm reduction model)** | **Women who accessed medical abortion without professional supervision or counseling** | **Women hospitalized for incomplete abortion or postabortion complications** | **General population or women of reproductive age regarding their knowledge and experience with medical abortion** | **Men whose sexual partners had a medical abortion** | **Women who had a legal abortion with misoprostol in contexts where abortion is permitted only in specific situations** | **Literature review** |
| 1 | **Lafaurie MM, Grossman D, Troncoso E, Billings D, Chávez Alvarado S, Maira G et al: *El Aborto con Medicamentos en América Latina. Las Experiencias de las Mujeres en México, Colombia, Ecuador y Perú*. Population Council y Gynuity Health Projects; 2005.** | Mexico, Colombia, Ecuador and Peru |  |  |  |  |  |  |  |  |
| 2 | **Lafaurie MM, Grossman D, Troncoso E, Billings D, Chávez S: Women's perspectives on medical abortion in Mexico, Colombia, Ecuador and Peru: A qualitative study. *Reproductive Health Matters* 2005,13:75-83 *** | Mexico, Colombia, Ecuador and Peru |  |  |  |  |  |  |  |  |
| 3 | **Billings, D: Misoprostol alone for early medical abortion in a Latin American clinic setting. *Reproductive Health Matters* 2004,12 (Suppl):57-64*** | Non-identified Latin American country |  |  |  |  |  |  |  |  |
| 4 | **Chávez S, Britt-Coe A: El aborto médico desde la perspectiva de un grupo de mujeres atendidas en un servicio atendido por consejeras en un distrito populoso de Lima. Lima: Promsex (working paper); 2002** | Peru |  |  |  |  |  |  |  |  |
| 5 | **Carril Berro E, López Gómez A: *Entre el Alivio y el Dolor: Mujeres, Aborto Voluntario y Subjetividad*. Montevideo: Trilce; 2008.** | Uruguay |  |  |  |  |  |  |  |  |
| 6 | **Briozzo L, Vidiella G, Rodríguez F, Gorgoroso M, Faundes A, Pons JE: A risk reduction strategy to prevent maternal deaths associated with unsafe abortion. *International Journal of Gynecology and Obstetrics* 2006,95:221-226*** | Uruguay |  |  |  |  |  |  |  |  |
| 7 | **Chaneton J, Vacarezza N: *La Intemperie y lo Intempestivo. Experiencias del Aborto Voluntario en el Relato de Mujeres y Varones*. Buenos Aires: Marea Editorial; 2011.** | Argentina |  |  |  |  |  |  |  |  |
| 8 | **Maroto Vargas, A: *Al Amparo de la Noche. La Ruta Crítica de las Mujeres que se Realizan Abortos Clandestinos en Costa Rica*. San José de Costa Rica: Asociación Colectiva por el Derecho a Decidir; 2010.** | Costa Rica |  |  |  |  |  |  |  |  |
| 9 | **Rance S. Entre ellos saben: Género y poder en relatos de mujeres bolivianas sobre sus experiencias con el aborto medicamentoso. Trabajo presentado en la Conferencia Latinoamericana sobre Prevención y Atención del Aborto Inseguro. Lima: CLACAI, 29 y 30 de junio de 2009** | Bolivia |  |  |  |  |  |  |  |  |
| 10 | **Zamberlin, N. y Gianni, M. C.: El circuito del misoprostol: un estudio de las respuestas a la demanda de medicamentos abortivos en farmacias privadas. Revista Medicina 2207(67) supl. I, pág. 67.** | Argentina |  |  |  |  |  |  |  |  |
| 11 | **Sherris, J, Bingham A; Burns MA, Girvin S, Westley E, Gomez, PI. Misoprostol use in developing countries: results from a multicountry study. International Journal of Gynecology and Obstetrics 2005; 88:76-81*** | Two non- identified countries in Latin America, one in Southeast Asia, and one in Africa. |  |  |  |  |  |  |  |  |
| 12 | **Barbosa R, Arilha M. The Brazilian Experience with Cytotec. Studies in Family Planning 1993 Jul-Aug; 24(4): 236-240*** | Brazil |  |  |  |  |  |  |  |  |
| 13 | **Vázquez S, Gutiérrez M.A, Calandra N, Berner E. El aborto en la adolescencia. En: Investigación sobre el uso de misoprostol para la interrupción del embarazo en adolescentes. Checa S. (compiladora). Realidades y Coyunturas del Aborto. Entre el derecho y la necesidad. Buenos Aires: Paidós; 2008. p. 277-297** | Argentina |  |  |  |  |  |  |  |  |
| 14 | **Faundes A, Santos LC, Carvalho M, Gras C. Post-abortion complications alter interruption of pregnancy with misoprostol. Advances in Contraception 1996; 12: 1-9*** | Brazil |  |  |  |  |  |  |  |  |
| 15 | **Troncoso E; Billings, D; Gómez Ponce de León R, Suárez C. Panorama del aborto con medicamentos: Resultados de diagnósticos en 5 países de América Latina en 2006. IPAS, Chapel Hill, 2007** | Bolivia, Brazil, Mexico, Panama and Nicaragua |  |  |  |  |  |  |  |  |
| 16 | **Pedrosa IL, Garcia TR. Não vou esquecer nunca!: A experiência feminina com o abortamento induzido. Revista latino-americana de enfermagem 2000; 8(6):50-58*** | Brazil |  |  |  |  |  |  |  |  |
| 17 | **Coelho HL, Texeira AC, Santos AP; Barros Forte E, Macedo Morais S, La Vecchia C, Togono G, Herxheimer A. Misoprostol and illegal abortion in Fortaleza, Brazil. The Lancet 1993; 341(8855):1261-1263*** | Brazil |  |  |  |  |  |  |  |  |
| 18 | [**Coêlho HL**](http://www.ncbi.nlm.nih.gov/pubmed?term="Coêlho HL"%5BAuthor%5D)**,**[**Teixeira AC**](http://www.ncbi.nlm.nih.gov/pubmed?term="Teixeira AC"%5BAuthor%5D)**,**[**Cruz Mde F**](http://www.ncbi.nlm.nih.gov/pubmed?term="Cruz Mde F"%5BAuthor%5D)**,**[**Gonzaga SL**](http://www.ncbi.nlm.nih.gov/pubmed?term="Gonzaga SL"%5BAuthor%5D)**,**[**Arrais PS**](http://www.ncbi.nlm.nih.gov/pubmed?term="Arrais PS"%5BAuthor%5D)**,**[**Luchini L**](http://www.ncbi.nlm.nih.gov/pubmed?term="Luchini L"%5BAuthor%5D)**,**[**La Vecchia C**](http://www.ncbi.nlm.nih.gov/pubmed?term="La Vecchia C"%5BAuthor%5D)**,**[**Tognoni G**](http://www.ncbi.nlm.nih.gov/pubmed?term="Tognoni G"%5BAuthor%5D)**. Misoprostol: The experience of women in Fortaleza, Brazil. Contraception 1994 Feb. 49(2):101*** | Brazil |  |  |  |  |  |  |  |  |
| 19 | **Costa SH, Vessey MP. Misoprostol and illegal abortion in Rio de Janeiro, Brazil. The Lancet 1993; 341(8855):1258-1261*** | Brazil |  |  |  |  |  |  |  |  |
| 20 | **Costa, SH. Commercial availability of misoprostol and induced abortion in Brazil. International Journal of Gynecology & Obstetrics 1998; 63(1 Suppl.):S131–S139*** | Brazil |  |  |  |  |  |  |  |  |
| 21 | **Pedroso D, de Campos Gomez E, Drezzet J. Abortamento previsto em lei em situacoes de violencia sexual. Perspectivas e experiencias das mulheres. En: Adesse L, Monteiro M. Magnitude do aborto no Brasil. Aspectos epidemiológicos e socio-culturais. Rio de Janeiro: IPAS, 2007** | Brazil |  |  |  |  |  |  |  |  |
| 22 | **Carril Berro E; López Gómez A. Varones y aborto voluntario: perspectivas de varones de distintas generaciones y niveles educativos. Informe técnico; 2008** | Uruguay |  |  |  |  |  |  |  |  |
| 23 | **Petracci M, Pecheny M, Capriati A, Mattioli, M. Varones, aborto y trayectorias socioafectivas según las experiencias y relatos de varones y mujeres de Buenos Aires, Argentina. Trabajo presentado en la VIII Reunión de Antropología del Mercosur: “Diversidad y poder en América Latina”. UNSAM, Buenos Aires, 2009** | Argentina |  |  |  |  |  |  |  |  |
| 24 | **Ouvinha Peres S, Heilborn ML. Cogitação e prática do aborto entre jovens em contexto de interdição legal: o avesso da gravidez na adolescência. Cad. Saúde Pública, 2006 Jul. 22(7):1411-1420*** | Brazil |  |  |  |  |  |  |  |  |
| 25 | **Diniz D, Corrêa M (coords). Aborto e saúde pública 20 anos de pesquisas no Brasil. Ministerio da Saude, Brasil, 2008** | Brazil |  |  |  |  |  |  |  |  |
| 26 | **Zamberlin N, Raiher S. Revisión del conocimiento disponible sobre experiencia de las mujeres con el uso del misoprostol en América Latina. Consorcio Latinoamericano contra el Aborto Inseguro y Centro de Estudios de Estado y Sociedad, 2010.** | Latin America |  |  |  |  |  |  |  |  |
| 27 | **Araujo, MJ. O uso do misoprostol pelas mulheres. En Direitos Reprodutivos no Brasil. Trilhas de Saúde das Mulheres, dos Direitos Sexuais e Dereitos Reprodutivos. Caderno 01, 2010. Rede Feminista de Saúde.** | Brazil |  |  |  |  |  |  |  |  |
